# Presentation of Lung Carcinoid Tumor as Post-obstructive Pneumonia

**DOI:** 10.7759/cureus.31859

**Published:** 2022-11-24

**Authors:** Benjamin Maruska, Abrahim N Razzak, Jose L Zepeda, Julia Novotny, Pinky Jha

**Affiliations:** 1 School of Medicine, Medical College of Wisconsin, Milwaukee, USA; 2 Internal Medicine, Medical College of Wisconsin, Wauwatosa, USA

**Keywords:** internal medicine, biopsy, tumor grade, carcinoid tumor, post-obstructive pneumonia, lung carcinoid tumor

## Abstract

Carcinoid tumors consist of neuroendocrine cells that produce amines, polypeptides, and prostaglandins. The majority of carcinoid tumors are found in the gastrointestinal system while a minority originate as pulmonary neoplasms. Among lung cancers, carcinoid tumors are rare, compromising 1-2% of lung malignancies in the United States. Lung carcinoid tumors are characterized into typical and atypical classifications. Typical lung carcinoid tumors are often lower grade, slower growing, and more well-defined than atypical tumors. Atypical tumors are also more likely to metastasize than their typical counterparts.

The patient presented in this article is a 35-year-old male with a history of recent hospital admission for pneumonia who presented with right chest pain. The patient was admitted eight days prior due to cough and acute hypoxemic respiratory failure secondary to post-obstructive pneumonia. During that admission, which totaled five days, he underwent a bronchoscopy and biopsy for a nodular right infrahilar opaque mass that appeared on computed tomography angiography of the chest. After the workup was negative, the patient was discharged. Three days later, he was re-admitted with continued chest pain. Biopsy results from the initial admission characterized the obstructing infrahilar mass as a carcinoid tumor with positive synaptophysin/ chromogranin stain and low proliferation (Mib1 < 2%). Following his discharge three days later, he was seen in follow-up by cardiothoracic surgery and underwent further imaging studies. Two months later, the patient underwent robotic right middle and lower bilobectomy. Pathologic analysis showed negative excised nodes and tumor margins.

Often, patients presenting with post-obstructive pneumonia are thought to have an underlying etiology that is purely infectious. This can lead to a delay in the discovery of the primary cause of the obstruction, and the underlying malignancy. Fortunately for this case, a biopsy was performed during the initial hospitalization, which led to a modification of his treatment plan early on in his second hospital stay after the tumor was characterized.

## Introduction

Carcinoid tumors are rare neuroendocrine tumors (NET) whose cells produce amines (serotonin, norepinephrine, dopamine), polypeptides (somatostatin, gastrin, chromogranin A), and prostaglandins. They also stain positive for potassium chromate (chromaffin) [1]. Carcinoid tumors most often originate in the gastrointestinal system but can also arise in the lung, with presenting symptoms of cough, hemoptysis, wheezing, and post-obstructive pneumonia (PNA) (especially if centrally located) [2]. The following report details a unique case of lung carcinoid tumor presenting as post-obstructive PNA.

This case has been previously presented at the American College of Physicians Wisconsin Chapter Scientific Meeting on September 9, 2022, at Glacier Canyon Conference Center, Wisconsin Dells, Wisconsin, United States of America.

## Case presentation

A 35-year-old male with a past medical history of gastroesophageal reflux disease, asthma, atrophic kidneys, and PNA presented with right chest pain.

Eight days before the patient's initial visit to our institution, he was admitted to an outside hospital with a cough and acute hypoxemic respiratory failure secondary to post-obstructive PNA. Upon admission at the outside hospital, a chest X-ray showed partial atelectasis of the right middle and right lower lobes. Subsequent chest computed tomography with angiography (CTA) revealed occluded bronchus intermedius with a mass-like, nodular right infrahilar opacity. Pulmonology was consulted, and a bronchoscopy was performed on hospital day four for a biopsy of the mass. Per infectious diseases recommendation, further workup was needed. This yielded a negative respiratory pathogen panel and respiratory culture (right lower lobe bronchoalveolar lavage) that grew gram-positive cocci. The patient was discharged on day five with a seven-day course of oral amoxicillin/clavulanic acid (Augmentin).

**Figure 1:**
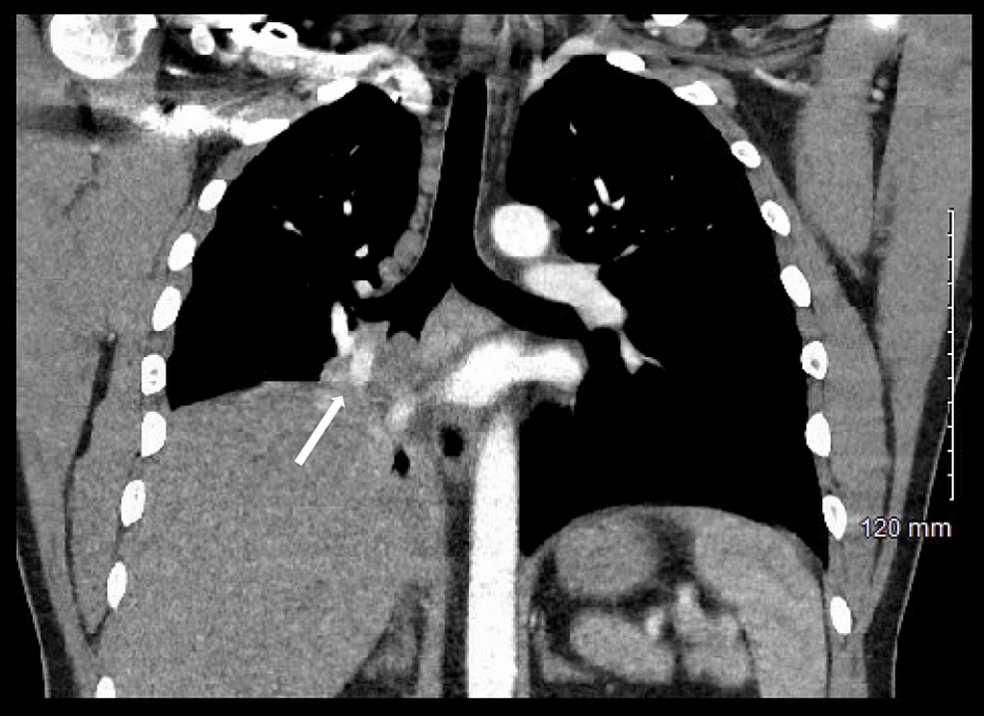
Coronal view of computed tomography angiography of the chest with contrast per the pulmonary embolism protocol, demonstrating persistent soft tissue density at the right hilum

**Figure 2:**
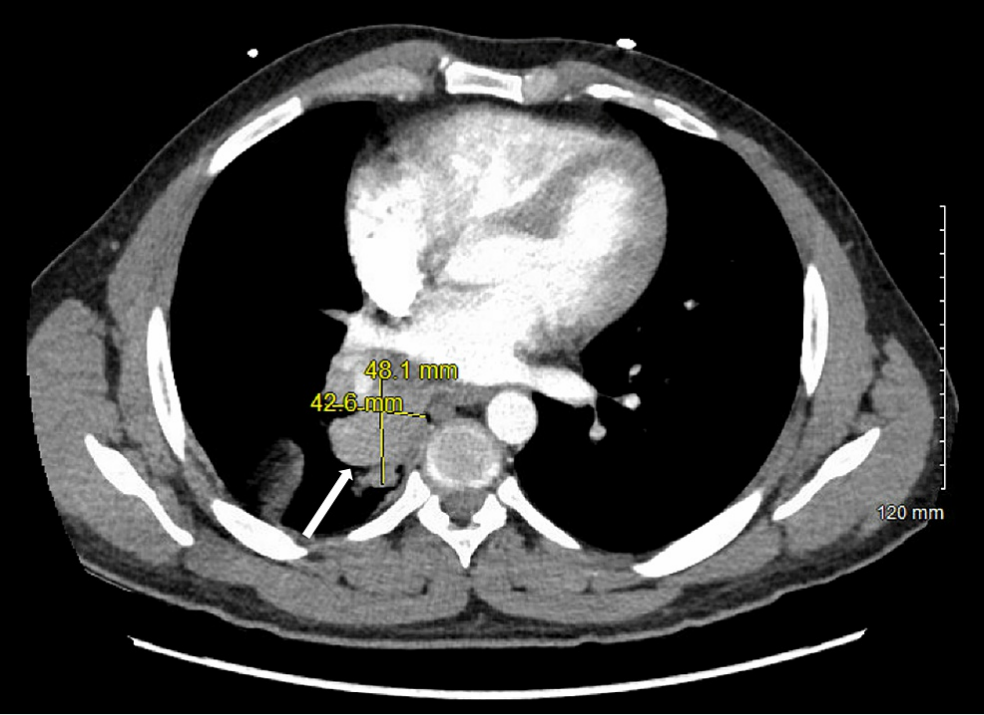
Axial view of computed tomography angiography of the chest with contrast per the pulmonary embolism protocol, demonstrating persistent soft tissue density at the right hilum indicative of an obstruction (42.6mm×48.1mm)

**Figure 3:**
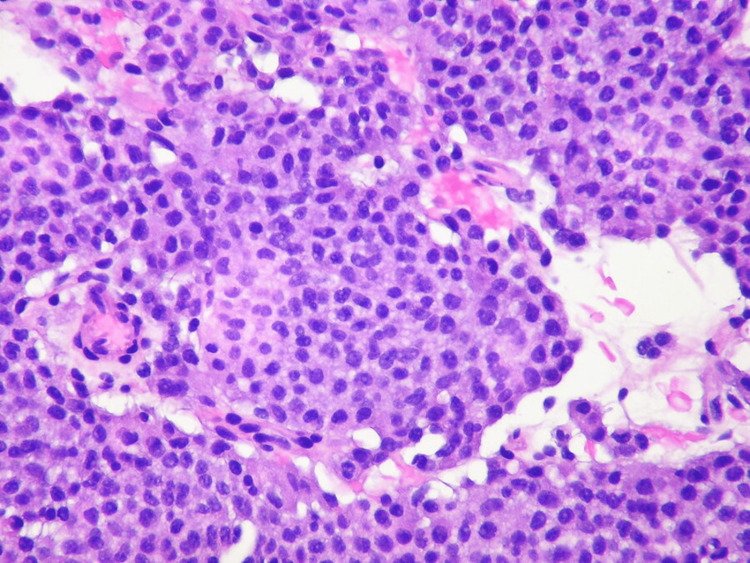
Sample pathological view of a typical carcinoid tumor with hematoxylin and eosin stain The view is exhibiting an insular/organoid growth pattern with small, uniform nuclei containing finely granular chromatin and non-prominent nucleoli. Image licensed under Attribution-ShareAlike CC-BY-SA 2.0 and permission obtained. Credits to Yale Rosen, M.D. for sample images. Source: [3]

**Figure 4:**
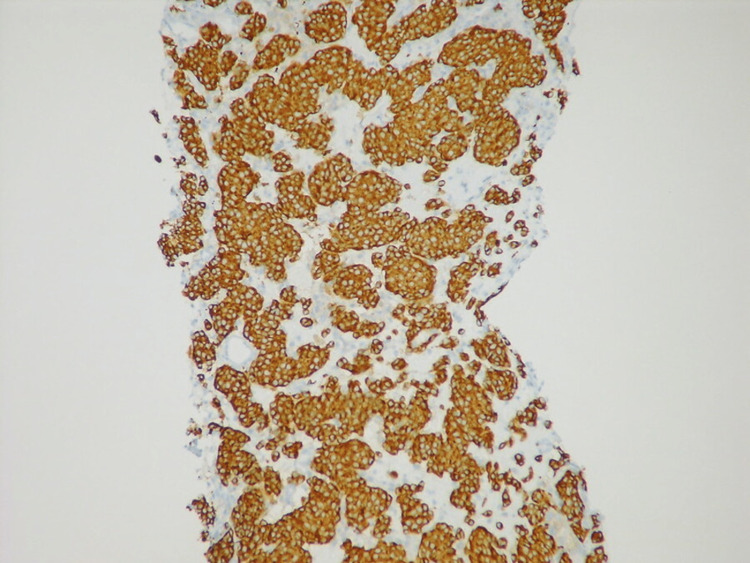
Sample pathological view of a typical carcinoid tumor with insular/organoid growth pattern Chromogranin immunostain is used in this view to mark tumors. Image licensed under Attribution-ShareAlike CC-BY-SA 2.0 and permission obtained. Credits to Yale Rosen, M.D. for sample images. Source: [3]

The patient was re-admitted to our institution with right-sided chest pain three days after being discharged from the outside hospital. In the emergency department, the patient was tachycardic at 118 BPM and had lab work significant for leukocytosis at 16.8×109/L (increased from 14.5×109/L at the time of the previous discharge). Upon admission, a cardiac ultrasound revealed no cardiac effusion and a normal left ventricular ejection fraction. CTA of the chest showed no acute pulmonary embolus, continued occlusion of the bronchus intermedius, and increased right lung consolidation since the prior admission (Figures 1, 2). Pulmonology was consulted, and a bronchoscopy was performed on hospital day one for the placement of three temporary stents. Pathology results from the biopsy taken during the previous admission characterized the obstructing mass as a carcinoid tumor with positive synaptophysin and chromogranin stains, and Mib1 <2% (low proliferation). Sample pathology images of typical carcinoid tumors are presented (Figures 3, 4) [3]. Cardiothoracic surgery was consulted for right lower lobe lobectomy, and hematology/oncology was contacted for outpatient follow-up. Computed tomography (CT) of the abdomen and pelvis on day two revealed no significant adenopathy or findings suggestive of metastatic disease. Magnetic resonance imaging of the brain on day three revealed no evidence of metastatic disease. The patient was discharged on hospital day three with a lidocaine patch and oxycodone for pain control. Albuterol nebulizer and acetylcysteine (Mucomyst) were also prescribed for airway clearance.

The patient underwent a positron emission tomography (PET) scan a month later, which showed several enlarged mediastinal lymph nodes with mild uptake and no other evidence of metastatic disease in the chest, abdomen, or pelvis. Shortly after this scan, pulmonology removed the stents that were placed during his second hospital stay. Two months after the PET scan, the patient underwent robotic right middle and right lower bilobectomy by cardiothoracic surgery. The patient tolerated the procedure well and was discharged three days after the operation. His one and two-week follow-up visits were significant for postoperative pain, which was eventually brought under control with gabapentin and methocarbamol. Final pathology results reported that all removed nodes and excised tumor margins were negative for malignancy.

## Discussion

Here we report a case of lung carcinoid tumor in a patient who was initially admitted to the hospital after presenting with cough, shortness of breath, and hypoxic respiratory failure secondary to post-obstructive PNA. The patient was readmitted three days after the initial hospital course for continued chest pain, and biopsy results came in for a carcinoid tumor.

The vast majority of carcinoid tumors occur in either the gastrointestinal tract (68%) or the lungs (25%) [4]. Among all lung cancer forms, carcinoid tumors are relatively rare, comprising 1-2% of all lung malignancies or approximately 2,000 to 4,500 new diagnoses per year in the United States [1,4]. The worldwide incidence of lung carcinoid tumors ranges from 0.2 to 2 per 100,000 people per year. Female and White patients are disproportionately more affected than male and Black patients respectively [2,4].

Carcinoid tumors are divided into two subclasses: typical (low grade) and atypical (high grade). Typical lung carcinoid tumors are roughly four times more common than atypical tumors. The average age of onset of typical lung carcinoid tumors is 45 years of age, approximately 10 years younger than that of atypical cases [5]. The tumor, node, metastasis classification system is used to stage lung carcinoid tumors. Typical lung carcinoid tumors are most commonly stage I when discovered; more than half of atypical cases are later stages at presentation [5].

Surgical resection with mediastinal lymph node sampling is the preferred treatment option for patients with low or intermediate-grade lung carcinoid tumors. There is a plethora of surgical techniques available that are chosen based on the size and location of each tumor. For small peripheral tumors, lung parenchyma can be spared via the utilization of sleeve resection [6]. Lobectomy is often the preferred approach in larger tumors, regardless of grade, especially if they are proximally located [7,8]. Mediastinal lymph node sampling is indicated at the time of surgery because 5-20% of typical lung carcinoid tumors and 30-70% of atypical lung carcinoid tumors metastasize into neighboring lymphatics [5,7]. Following resection of typical lung carcinoid tumors 5- and 10-year survival rates are 87-100% and 82-87%, respectively. Analogous 5- and 10-year survival rates of atypical lung carcinoid tumors are 30-95% and 35-56% [1,4,8].

The majority of patients with post-obstructive PNA secondary to malignancy undergo symptomatic treatment of respiratory and infectious symptoms with multiple rounds of antibiotics. This often leads to a delay in the discovery of the primary cause of the obstruction, the underlying malignancy. Fortunately for this case, a biopsy was performed during the initial hospitalization, which led to a modification of his treatment plan early on in his second hospital stay after the tumor was characterized.

## Conclusions

This case provides a compelling reminder of how careful consideration of a differential diagnosis leads to optimal patient outcomes. Following his initial presentation, this patient’s infectious symptoms were recognized and promptly treated with antibiotics. Fortunately, the diagnostic process did not stop there. Additional evaluation of the obstructive etiology causing the infectious symptoms led to further imaging, biopsy, and eventual mass characterization. The results of this thorough diagnostic process led to the discovery of the malignancy while it was still in the early stages, which remains an important prognostic factor in patients with lung cancer. Subsequent effective collaboration between medicine, pulmonology, cardiothoracic surgery, oncology, and pathology led to timely definitive treatment of the underlying pathology.
